# Role of subnetworks mediated by $\hbox {TNF}\alpha$, IL-23/IL-17 and IL-15 in a network involved in the pathogenesis of psoriasis

**DOI:** 10.1038/s41598-020-80507-7

**Published:** 2021-01-26

**Authors:** Rakesh Pandey, Yusur Al-Nuaimi, Rajiv Kumar Mishra, Sarah K. Spurgeon, Marc Goodfellow

**Affiliations:** 1 College of Engineering, Mathematics and Physical Sciences, University of Exeter, Exeter, UK; 2 Department of Dermatology, Royal Devon and Exeter Hospital, Exeter, UK; 3 Institute of Science and Technology Austria, Am Campus 1, Klosterneuburg, Austria; 4 Department of Electronic and Electrical Engineering, University College London, London, UK; 5 Living Systems Institute, University of Exeter, Exeter, UK; 6 Wellcome Trust Centre for Biomedical Modelling and Analysis, University of Exeter, Exeter, UK; 7 EPSRC Centre for Predictive Modelling in Healthcare, University of Exeter, Exeter, UK; 8 Present Address: Bioinformatics, Mahila Mahavidyalay, Banaras Hindu University, Varanasi, India; 9 Present Address: iOligos Technologies Private Limited, Noida, India

**Keywords:** Systems biology, Computer modelling, Psoriasis

## Abstract

Psoriasis is a chronic inflammatory skin disease clinically characterized by the appearance of red colored, well-demarcated plaques with thickened skin and with silvery scales. Recent studies have established the involvement of a complex signalling network of interactions between cytokines, immune cells and skin cells called keratinocytes. Keratinocytes form the cells of the outermost layer of the skin (epidermis). Visible plaques in psoriasis are developed due to the fast proliferation and unusual differentiation of keratinocyte cells. Despite that, the exact mechanism of the appearance of these plaques in the cytokine-immune cell network is not clear. A mathematical model embodying interactions between key immune cells believed to be involved in psoriasis, keratinocytes and relevant cytokines has been developed. The complex network formed of these interactions poses several challenges. Here, we choose to study subnetworks of this complex network and initially focus on interactions involving $\hbox {TNF}_{\alpha }$, IL-23/IL-17, and IL-15. These are chosen based on known evidence of their therapeutic efficacy. In addition, we explore the role of IL-15 in the pathogenesis of psoriasis and its potential as a future drug target for a novel treatment option. We perform steady state analyses for these subnetworks and demonstrate that the interactions between cells, driven by cytokines could cause the emergence of a psoriasis state (hyper-proliferation of keratinocytes) when levels of $\hbox {TNF}_{\alpha }$, IL-23/IL-17 or IL-15 are increased. The model results explain and support the clinical potentiality of anti-cytokine treatments. Interestingly, our results suggest different dynamic scenarios underpin the pathogenesis of psoriasis, depending upon the dominant cytokines of subnetworks. We observed that the increase in the level of IL-23/IL-17 and IL-15 could lead to psoriasis via a bistable route, whereas an increase in the level of $\hbox {TNF}_{\alpha }$ would lead to a monotonic and gradual disease progression. Further, we demonstrate how this insight, bistability, could be exploited to improve the current therapies and develop novel treatment strategies for psoriasis.

## Introduction

Psoriasis is a prevalent chronic inflammatory skin disease which affects people of all ages and ethnicities^1^. Chronic plaque psoriasis (also known as psoriasis vulgaris) is the commonest form and occurs in 90% of cases^2^. It has a characteristic appearance that consists of well demarcated, inflamed plaques covered with silvery-white scales. The outer-most layer of the human skin (epidermis) thickens in these lesions due to increased proliferation and abnormal differentiation of keratinocytes, which are the predominant cells of the epidermis. The severity of psoriasis can range from the involvement of small, isolated areas to covering the majority of patients’ skin. In the case of mild disease, topical treatments are prescribed whereas phototherapy (using ultraviolet light), systemic and biological therapies are used for moderate-to-severe cases. Although these therapies are often successful, response varies from patient to patient, and it is therefore crucial to better understand why these treatments work in some cases and not others.

Psoriasis had previously been described as a primary disorder of keratinocyte proliferation and differentiation. However, advances in genetic and immunological techniques have led to improvements in our understanding of psoriasis, which is now widely recognised as a complex and multi-factorial, immune-mediated inflammatory disease^3–5^. Psoriasis can therefore be thought of as a multi-scale dynamic disease arising as a manifestation of the dysregulated interaction between keratinocytes and the immune system. Given this complexity and in order to understand how the disease state arises, research strategies involving experimental and mathematical modelling of these processes are essential^6^. As such, several in vitro and in vivo models have been proposed to study its underlying causes^7^ along with the use of in silico models to understand healthy and psoriatic epidermic growth. These in silico models can be classified into two groups: (1) agent-based^8–12^, and (2) ordinary differential equation (ODE) based^13–15^. The agent based models could simulate the non-lesional and psoriatic keratinocyte compartment of the epidermis as well as the UVB (Ultraviolet B) intervention for plaque resolution. In this method, each cell is represented by a software agent and each agent has a rule set that determines the behavior. The rule set also determines the interaction of agents with their neighbours and that can be used to model the organisation of multicellular aggregates^8^. An ODE model developed by Valeyev et al.^13^ considered interactions between immune cell populations based on their cytokine production profiles and informed the mechanism of inflammation in human skin. Mathematical models for the pathogenesis of psoriasis have been developed by Roy et al.^14,15^ involving the densities of immune cells and keratinocytes. They have considered the hyper-proliferation of keratinocytes as one of the precursors of psoriasis. Further, they demonstrated a method for stable control of the keratinocyte population using negative feedback. In a recent study, they have demonstrated the role of Th1 and Th2 cells in the pathogenesis of psoriasis using an ODE based mathematical model^16^.

**Figure 1:**
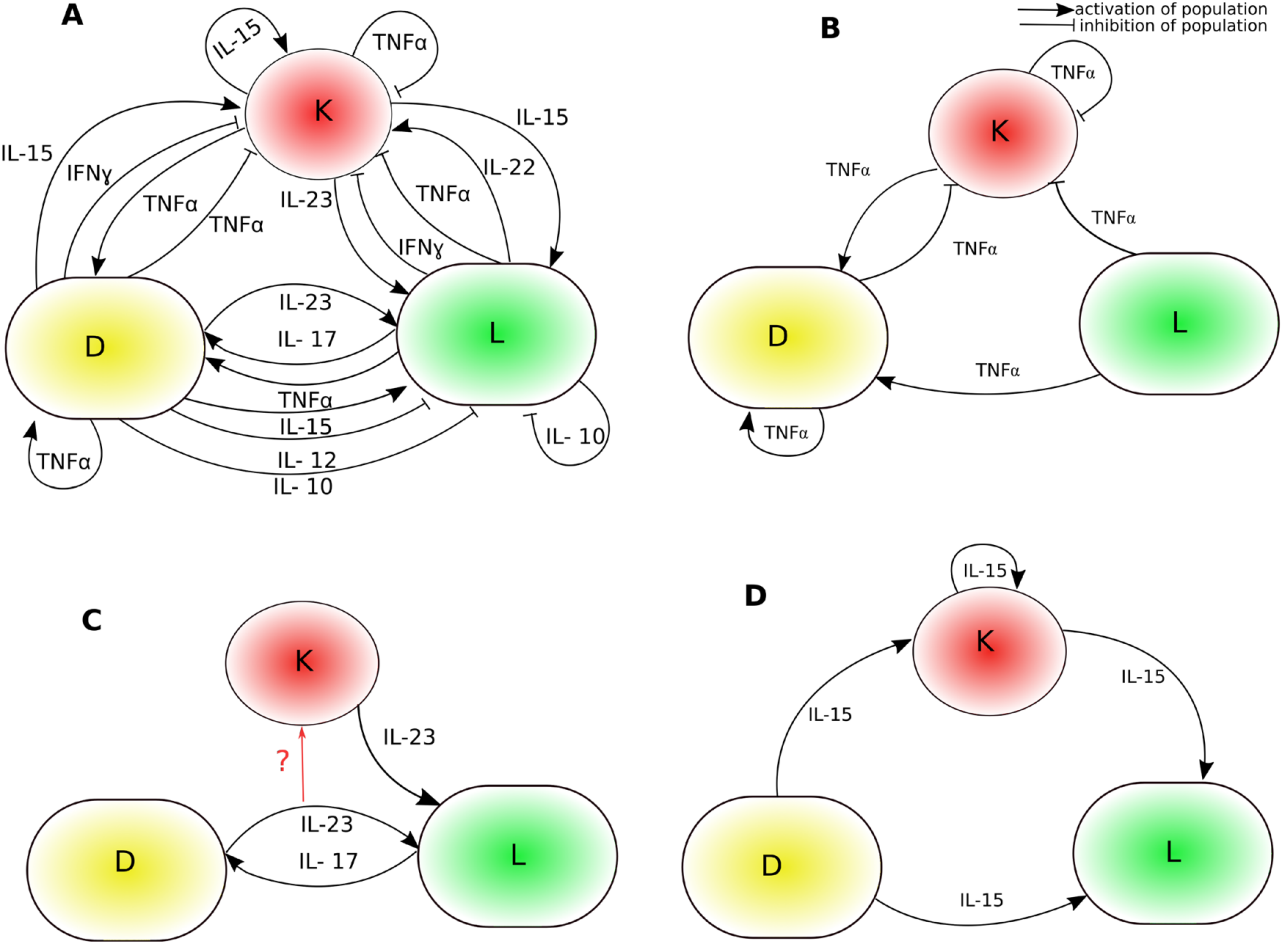
(**A**) A schematic diagram explaining cytokine mediated interactions among the cell types predominantly implicated in psoriasis. Here, L denotes the population of T-cells and for simplicity a single pool is considered. The symbol D represents matured dendritic cells(DCs). Note that the primary role of immature DCs is in antigen detection, uptake and processing whereas mature DCs are believed to function in antigen presentation and cytokine production^17^. The symbol K represents the population of keratinocytes. Arrows show an increase in population and lines with a bar end denote inhibition of the population. (**B**) A subnetwork of the network (**A**) involving only $\hbox {TNF}_{\alpha }$. (**C**) Cytokine IL-17 and IL-23 mediated subnetwork of the network. Here, a red colored arrow represents the net effect of the dendritic and T-cell population on keratinocytes. The question mark indicates the lack of clarity about this signal which could be either inducing or inhibiting. (**D**) The subnetwork involving cytokine IL-15 alone.

In order to further our understanding of the role that immune cells and cytokines play in the pathogenesis of psoriasis, we recently synthesised current evidence into a mathematical model of the network of cytokine-mediated interactions between key immune cells (T-cells and dendritic cells) and keratinocytes^18^. The inclusion of keratinocytes was crucial to enable the diseased and healthy psoriasis phenotypes to be modelled. Within this framework, we demonstrated the existence of healthy and diseased states, as well as the potential for cytokines to behave as finite-time controllers of the state of the system. This model therefore offers the potential to advance our understanding of the mechanisms underlying the success or failure of biological treatments. A challenging task in terms of analysis, however, is the complexity of the system, as interactions between cells are mediated by many different cytokines.

To further our insight into this system here we identify specific cytokines those mediate subnetworks of the network formed of key immune cells and keratinocytes. Such subnetworks could be particularly important for governing system dynamics^19^, but also allow a systematic analysis of how they may mediate transitions between healthy and psoriatic states. In particular, we focus on sub-networks mediated by cytokines $\hbox {TNF}_{\alpha }$, IL-17/IL-23 axis and IL-15. Selection of $\hbox {TNF}_{\alpha }$ and IL-17/IL-23 axis is inspired by biologics either approved by the FDA (Food and Drug Administration) or under clinical trials targeting them^20–22^. However, to date there is no psoriasis treatment directly targeting IL-15. Indeed, evidence linking IL-15 to psoriasis pathogenesis is less abundant than the other cytokines considered above (though see, for example^23–25^). Nevertheless, the interactions between cell types relevant for psoriasis that we identified do involve IL-15 and our research indicates that IL-15 links all 3 cell types considered. It is therefore interesting to understand the role that it plays in regulating the dynamics of this network. In addition, the structure of the subnetwork mediated by IL-15 resembles the well known coherent feed forward loop motif observed and analysed in the context of gene regulatory networks .We study the asymptotic behaviour of these subnetworks and demonstrate the potential for each of these cytokines to mediate transitions between healthy and psoriasis states. We find that increases in levels of $\hbox {TNF}_{\alpha }$ and IL-23/IL-17 lead to a high keratinocyte state, which is in line with their inhibitors being used as treatments for psoriasis. Our analysis further suggests that these cytokines exhibit different dynamic routes to psoriasis. Hyper-proliferation of keratinocytes due to increases in the level of IL-23/IL-17 can occur via a bistable regime in which two steady state populations of keratinocytes is possible. A similar behaviour also occurs for the subnetwork involving cytokines IL-15. In contrast, the progression of psoriasis in the subnetwork formed by $\hbox {TNF}_{\alpha }$ is found to occur without bistability, instead giving rise to a monotonic increase in the keratinocyte population. Due to bistability, we expect the possibility of a quicker disease progression mediated by IL-17/IL-23 and IL-15 than $\hbox {TNF}_\alpha$. Thus, we may observe a faster onset of recovery for biologics targeting IL-17/IL-23 and IL-15 in compared to $\hbox {TNF}_\alpha$ inhibitors. These results are consistent with the observation that IL-17 inhibitors act quickly and efficiently than $\hbox {TNF}_\alpha$ inhibitors^21,22,26^. However, the better clinical response of IL-17 is not attributed to bistability yet. Further, we discuss the relevance of these findings for the future development of treatments for psoriasis.

## Results

### Dynamics of the subnetwork mediated by the $\hbox {TNF}_{\alpha }$

We first study the dynamics of the subnetwork that emerges due to interactions mediated by $\hbox {TNF}_{\alpha }$ (Fig. 1B; Eq. 7). Although we have greatly reduced the complexity of the system, six parameters remain which determine its dynamics.

**Figure 2:**
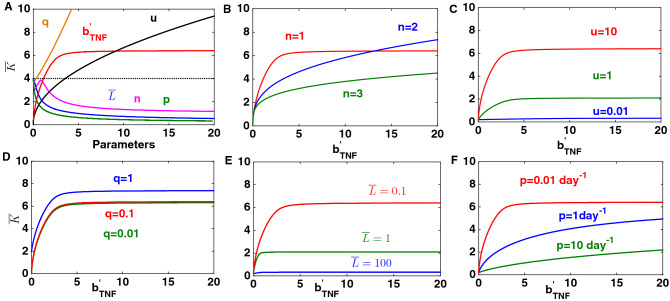
Steady state behavior of the subnetwork mediated by $\hbox {TNF}_{\alpha }$ (Fig.1B). (**A**) Steady state response curve of the keratinocyte population for varying model parameters of the subnetwork. The “parameters” on the abscissa, represent $b^{'}_{TNF}, p, q, n, {\bar{L}}$, and *u* and the likely variation is shown by different coloured lines. (**B**–**F**) The response curve of the keratinocytes as a function of the varying level of $\hbox {TNF}_{\alpha }$ can be refined and adjusted by changing other parameters. Effect of changes in parameters: Hill coefficient (*n*), *u*, *q*,${\bar{L}}$, and *p* on the steady state response of keratinocyte population to varying $b^{'}_{TNF}$ (level of $\hbox {TNF}_{\alpha }$), respectively. The default parameters are: $b^{'}_{TNF}=1$, $p=0.01$ day$^{-1}$, $q=0.1$ day$^{-1}$, ${\bar{L}}=0.1$, $u=10$, $n=1$, $\mu _K=0.5$ day$^{-1}$, and $\mu _D=0.2$ day$^{-1}$. The initial condition is ${\bar{K}}=4$ and ${\bar{D}}=2$ throughout.

The steady state behaviour of the subnetwork for varying model parameters is investigated using bifurcation analysis techniques of dynamical systems and the result is shown in Fig. 2. We study the effect of varying parameters independently in the first instance, focussing on parameters that directly modulate the keratinocyte population (such as *q*), embody the cytokine effect ($b^{'}_{TNF}$) and the effect of changes in T-cell numbers (${\bar{L}}$). We observe that an increase in the modulatory effect of $\hbox {TNF}_{\alpha }$ i.e. high level of $TNF_{\alpha }$ represented by $b^{'}_{TNF}$ could lead to increase in the population of keratinocytes and therefore to psoriasis as the keratinocytes are observed to be hyper-proliferative in psoriatic skin^3,27,28^. The keratinocyte population would also increase with increase in the effective rate of migration of keratinocytes denoted by *q*. Also, psoriasis would develop when the strength of the modulatory effect of dendritic cells on other modelled cells is stronger than that of keratinocytes, represented by a large value of *u* (Fig. 2A). The large vale of *u* (= $\frac{k_D}{k_K}$) suggests that the impact of the keratinocyte cell population on dendritic cells and its own population would be stronger than the impact of the dendritic cell population.

In contrast to the above, the keratinocyte population decreases with increase in the steady state population of T-cells (${\bar{L}}$), Hill coefficient (*n*), and effective rate of migration of dendritic cells towards the lesion (*p*) (Fig. 2).

Based on the known mode of action of anti-TNF biologics, we use Fig. 2A to define healthy and psoriasis states. It is henceforth assumed that a value of ${\bar{K}}$ above 4 is considered as a psoriasis state. In fact, a four-fold increase in the number of keratinocytes compared to healthy skin has been previously suggested^13^. We observe a gradual and monotonic increase in keratinocytes therefore these results suggests that this subnetwork would lead to a gradual progression of psoriasis.

We extend this analysis by considering the interplay between $b^{'}_{TNF}$ and the other parameters in Fig. 2B–F. Figure 2B demonstrates that decreasing the value of the Hill coefficient, *n* in Eq. (7), causes keratinocyte levels to have a higher sensitivity to increases in $b^{'}_{TNF}$. This sensitivity would also increase with increase in the effective rate of inward migration of keratinocytes (Fig. 2B) and the modulatory effect of the dendritic cell population being more effective than that of the keratinocyte population (Fig. 2C). Nonetheless, this sensitivity would be decreased by increasing the steady state level of T-cells (${\bar{L}}$) and the inward effective rate of migration of the dendritic cell population (*p*) (Fig. 2E, F). Here, $k_D$ and $k_K$ are assumed to have the properties of a dissociation constant in a Hill function. These results suggest that the steepness in increase or decrease in the population of keratinocytes in response to varying level of $\hbox {TNF}_{\alpha }$ could be modified by changing other model parameters. In other words, the sensitivity of the increase or decrease in the keratinocyte population to fluctuating level of $\hbox {TNF}_{\alpha }$ would be determined by other model parameters.

### Dynamics of the IL-23 and IL-17 mediated subnetwork

This subnetwork is mediated by the widely known “IL-23/IL-17” axis which has been extensively explored for developing a treatment for psoriasis^22,29,30^. This subnetwork is formed of two feedforward loops between dendritic and T-cells, the output of which determines the dynamics of the population of keratinocytes (Fig. 1C). Along-with studying the asymptotic behavior of this subnetwork, we wish to determine whether the net effect of the output of this axis on the population of keratinocytes is enhancing or inhibitory. We performed a steady state analysis of the subnetwork equations, accounting for either an activating (case 1) or inhibiting (case 2) effect of dendritic and T-cells on the population of keratinocytes (governed by Eqs. 9 and 11, respectively).

**Figure 3:**
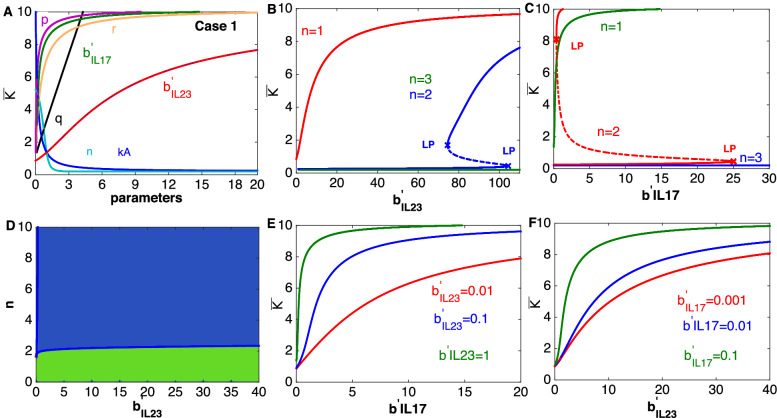
A characteristic steady state functioning of the subnetwork mediated by “IL-23/IL-17” axis in case 1. (**A**) Steady state response curve of the keratinocyte population for changing model parameter values of the subnetwork (Fig. 1C). (**B**,**C**) Response of the keratinocyte population to changes in the level of IL-23 and IL-17, represented by $b^{'}_{IL23}$ and $b^{'}_{IL17}$, respectively could be non-graded and non-monotonic, in fact bistable. Dotted lines correspond to the unstable steady state bounded by saddle node bifurcations denoted by LP. (**D**) The separation of the two parameter spaces formed by *n* and the level of IL-23 in Monostable (green) and bistable (blue) regions. (**E**) The current level of IL-23 dependent sensitivity on the increase in the population of keratinocytes for varying level of IL-17. (**F**) The IL-17 level dependent sensitivity of the increase in the population of keratinocytes in response to increase in the level of IL-23. The default parameters are: $b^{'}_{IL23}=1, b^{'}_{IL17}=0.01$, $b_{net} =5$, $p=0.01$ day$^{-1}$, $q=0.1$ day$^{-1}$, r = 0.01, n = 1, $\mu _L=0.5$ day$^{-1}$, $\mu _K=0.5$ day$^{-1}$, $\mu _D=0.2$ day$^{-1}$, and $k_A=1$. The initial condition is ${\bar{L}}={\bar{D}}=2$ and ${\bar{K}}=4$. *LP* limit points (saddle node bifurcations).

A steady state characteristic behavior of this subnetwork in case 1 is shown in Fig. 3 and Fig. S1A–L. Figure 3A suggests that the keratinocyte population increases with increase in the level of either of the cytokines, IL-23 and IL-17, represented by parameters $b^{'}_{IL23}$ and $b^{'}_{IL17}$, respectively. However, the increase is more sensitive to changes in IL-17 compared to IL-23 (Fig. 3A) . The keratinocyte population also increases with increase in the parameters *p*, *q* and *r* representing the effective rate of migration of dendritic, keratinocytes and T-cells, respectively towards the lesion. Increases in the parameter $k_A$ and *n* (representing the effective dissociation constant and Hill coefficient, respectively for the combined effect of IL-23 and IL-17) leads to a decrease in the population of keratinocytes. Note that changes in the above mentioned parameters result in either increase or decrease in the population of keratinocytes but monotonically. Therefore, these results suggest that for some parameter combinations, changes in the model parameters could lead to a gradual increase or decrease in the keratinocyte population.

Interestingly, the changes in the modulatory effect of IL-23 ($b^{'}_{IL23}$) or IL-17 ($b^{'}_{IL17}$) could lead to a pair of saddle-node bifurcations, and hence a region of bistability (Fig. 3B,C) especially for $n>1$. The bistable region is bounded by the saddle node bifurcations, $74.5<b^{'}_{IL23}<104.3$ and $0.4<b^{'}_{IL17}<25$ for the given parameter combinations and denoted by LP in the figure. In fact, there is a large region of bistability in the two parameter spaces of *n* vs $b^{'}_{IL23}$ and *n* vs $b^{'}_{IL17}$ as depicted in Fig. 3D and Fig. S1K,L. In the bistable region, two stable and an unstable steady state, shown by the solid and dotted line in Fig. 3B,C, respectively coexist. The bifurcation points separate the system’s response into two distinct regions—low and high keratinocyte populations. The meaning of the bistability will be further explained in the next section and Fig. 5. Note that, there is $\approx$ 4 fold difference in the low and high keratinocyte levels similar to the previous subnetwork (Fig. 1B). Also the high and low level of keratinocytes correspond to a psoriatic (diseased) and healthy state, respectively.

Our analysis suggests that the increase in the population of keratinocytes in response to the increase in the level of IL-17 would be sensitive to the present level of IL-23 (Fig. 3E). In fact, if the present level of IL-23 is comparatively high then the changes in keratinocyte population would be more sensitive to the changes in the level of IL-17 (Fig. 3E). Similarly, the present level of IL-17 would determine the sensitivity of the increase in the population of keratinocytes in response to the increase in the level of IL-23 (Fig. 3F). Like in panel E of Fig. 3 , a high present level of IL-17 ensures comparatively high sensitivity of increases in keratinocytes, in response to changes in the level of IL-23.

**Figure 4:**
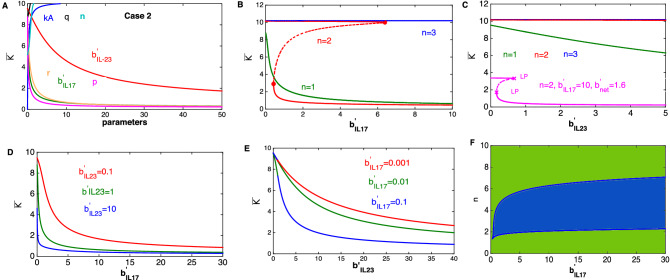
The signal response curve for the subnetwork mediated by IL-17 and IL-23 (Fig. 1C) in the case 2 scenario. (**A**) Steady state population of keratinocytes for varying model parameters. (**B**) Bistable response of keratinocyte population for changes in the level of IL-17. (**C**) For parameter combinations as in Fig. 3, bistability would not be observed. However, changing $b^{'}_{IL17}=10$, $b^{'}_{net}=1.6$ and n = 2 could lead to a bistable behavior. (**D**) Effect of the present level of IL-23 on the steady state response of the keratinocyte population for changing levels of IL-17. (**E**) Modulatory effect of the present level of IL-17 on the steady state response of the keratinocyte population for varying levels of IL-23. (**F**) The separation of the two parameter space spanned by the level of IL-17 and the Hill coefficient (n) in monostable (green) and bistable (blue) regions. Model parameters as in Fig. 3 except in (**C**) where $b^{'}_{IL17}=10$, $b^{'}_{net}=1.6$ and n = 2.

The steady state behavior of the subnetwork in case 2 is shown in Fig. 4. As shown in Fig. 4A, in contrast to case 1, the population of keratinocytes decreases with an increase in the level of IL-17 ($b^{'}_{IL17}$) and IL-23 ($b^{'}_{IL23}$) as well as with increase in the effective rate of migration of the cell populations (represented by *p*, *q*, and *r*). However, the high sensitivity to changes in the level of IL-17 compared to that of in the level of IL-23, is preserved as the population of keratinocytes decreases faster with increase in IL-17. Therefore, our analysis suggests that the proliferation of keratinocytes would be more sensitive to changes in the level of IL-17 than to IL-23 (Figs. 3A, 4A). Because of this, we argue that an IL-17 blocker could potentially be more effective than an IL-23 inhibitor.

The population of keratinocytes increases with increase in the parameter $k_A$ and *n*, which is opposite to what is observed in case 1. Therefore, the response to changes in the level of IL-17 and IL-23 in cases 1 and 2 are antagonist to each other (Figs. 3A, 4A) as expected.

In addition, compared to IL-17 and IL-23, the increase in the keratinocyte population is less sensitive to changes in the net population of dendritic and T-cells for small values of $b_{net}$ (Fig. S1B, G). For large values of $b_{net}$, the keratinocyte population increases almost linearly (Fig. S1B,G). This highlights the multi-faceted impact of changes in the cytokine levels in comparison with the effects of direct changes in the cell population.

We observe that case 2 could also give rise to a region of bistability in response to the changes in the level of IL-17 and IL-23 (Fig. 4B,C). However, in this case, for low levels of IL-23 and IL-17 the population of keratinocytes would be very high which is opposite to the case 1. In this case too, the region of bistability is large (Fig. 4F, Fig. S2L–N).

As with case 1, the decrease in the population of keratinocytes in response to increase in the level of IL-17 is sensitive to the present levels of IL-23 (Fig. 4E). In fact, it decreases faster at high level of IL-23 in response to the increase in the level of IL-17. Similarly, the population of keratinocytes decreases faster if the present level of IL-17 is high in response to an increase in IL-23 (Fig. 4F). These results suggest that although, in case 2, the population of keratinocytes decreases with increase in the level of IL-17 and IL-23, as opposed to case 1, the sensitivity of decrease (increase in case 1) would still be determined by the present level of IL-23 and IL-17, respectively.

Our analysis demonstrates that the net effect of the populations of dendritic and T-cells on keratinocytes would be activating to be consistent with the current knowledge about IL-17. The IL-17 family of cytokines have been suggested to be most relevant to the pathogenesis of psoriasis because of their action on keratinocytes and the increased level within psoriatic lesions^31,32^. Also, higher serum and lesional levels of a member of this family, IL-17A, in psoriasis patients compared to controls have been reported which supports the role of IL-17 A in this disease^31,33^. In addition, the use of IL-17 blockade (ixekizumab) has shown a significant reduction in keratinocyte proliferation and infiltration of T-cells and dendritic cells into the dermis and epidermis compared to the baseline^34^. Therefore, the hypothesis of case 1 is most likely in in vivo conditions.

### Dynamics of the subnetwork involving IL-15

**Figure 5:**
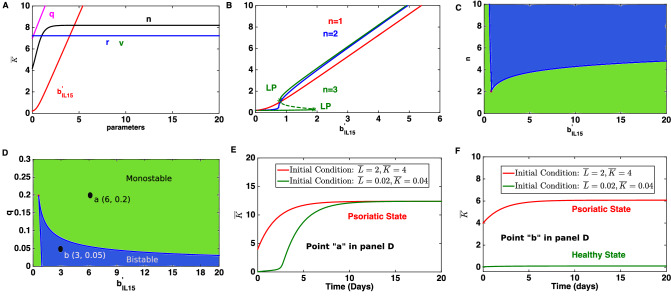
A signal response curve for the subnetwork involving cytokine IL-15 (Fig. 1D). (**A**) Changes in the stead state population of keratinocytes in response to changes in the model parameters. (**B**) The hyper-proliferation of keratinocytes can occur via a bistable regime in response to varying level of IL-15. In that regime the steady state keratinocyte population can exist in two distinct states: low and high. The low steady state population corresponds to a healthy population and the higher one corresponds to a psoriatic state. (**C**) The separation of bistable (blue) and monostable (green) regions in the two parameter spaces formed by the level of IL-15 and Hill coefficient (n). (**D**–**F**) A demonstration of typical trajectories of the keratinocyte population in monostable and bistable regions. While the point “a” is in monostable region, the point “b” lies in the bistable region. The model parameters are: $b^{'}_{IL-15}=4, v=0.1,r=0.01, q=0.1, n=1,\mu _L=0.5$ and $\mu _K=0.5$. The initial condition is ${\bar{L}}=2$ and ${\bar{K}}=4$.

This subnetwork is formed of a coherent feed-forward loop and self activation. Following the mathematical modelling frame work discussed above the population dynamics of this subnetwork (Fig. 1D) is governed by Eq. (14). The steady state response of the keratinocyte population to varying model parameters independently is shown in Fig. 5A. We observe that the increase in the level of IL-15, denoted by the large value of $b^{'}_{IL-15}$ would lead to increased keratinocyte population and therefore a psoriatic state. Similarly, increase in the parameter *q* and *r* representing the effective rate of migration of keratinocytes and T cells, respectively towards the lesion would result in psoriasis.

The keratinocyte population would also increase with increase in the parameter $v (=\frac{k_d}{k_k})$ which compares the strength of the effect caused by dendritic cells and keratinocytes on other modelled cell types (Fig. 5A). A large value of *v* denotes that the effect of dendritic cells on other modelled cell types should be stronger than that of the keratinocytes. The keratinocyte population would also increase with increase in the parameter *n*, defining the Hill coefficient characteristic (Fig. 5A). We analyse how other model parameters affect the steady-state response of the keratinocyte for varying levels of IL-15 (Fig. 5B, Fig. S3A–C). A steady state analysis of Eq. (14) shows that the population of keratinocytes could increase via a bistable regime (Fig. 5B). It also suggests that the subnetwork would not show bistable behavior if the Hill coefficient $n < 3$ (Fig. 5B). The exact steady state population of K in the bistable regime could be determined by the initial condition i.e. initial population of T-cells and keratinocytes. As observed with the subnetworks, the high value of *K* corresponds to a psoriatic state whereas a low value represents a healthy state since the keratinocytes are observed to be hyper-proliferative in psoriatic skin^3,27,28^. These results suggest that an increase in the intracellular IL-15 level could lead to a large increase in the keratinocyte population and therefore to a psoriatic state. The increase in the level of IL-15 could be caused by several internal and/or external factors.

Figure 5C,D suggest that this bistable phenomena is very robust as the system is bistable in a large region of the two parameter spaces. Examples of dynamics in the monostable and bistable region are shown in Fig. 5E,F. Note that in the bistable region, the system reaches two distinct steady states with the same parameter combination for different initial conditions.

In order to find the source of bistability in this subnetwork, we remove the auto activation of keratinocytes. It leads to the following modification to Eq. (13)

1 $$\begin{aligned} \begin{aligned} \frac{{\mathrm {d}}{\bar{K}}}{{\mathrm {d}}t}&= \frac{a_K +b_{IL-15}D^*}{k_K}-\mu _{K} {\bar{K}} \\ \frac{{\mathrm {d}}{\bar{L}}}{{\mathrm {d}}t}&= \frac{a_L}{k_K}+\frac{b_{IL-15}D^*}{k_K}\frac{{\bar{K}}^n }{1+{\bar{K}}^n}-\mu _L {\bar{L}} \end{aligned} \end{aligned}$$

Now, there is only one steady state: $L_S= \frac{a_L+b_{IL-15}D^* \frac{K_{S}^n}{1+K_{S}^n}}{\mu _L k_K}, K_S=\frac{a_K+b_{IL-15}D^*}{\mu _K k_K}$.

Here we observe that the subnetwork does not show bistability in the absence of the self activation of keratinocytes. This is not surprising as the presence of a positive feedback loop is a necessary condition for a reaction network to exhibit bistability^35–37^. Therefore, our results suggest that the source of bistability in this subnetwork would be the self activation loop of keratinocytes.

### A novel therapy for treating Psoriasis by exploiting bistability

The present study suggests that the subnetworks involving the IL-23/IL-17 axis and IL-15 cytokine could cause psoriasis via bistable regimes, we explore if this behavior can be exploited in order to find a new treatment option. Our approach is demonstrated for the subnetwork formed by IL-15.

In the model, an IL-15 level corresponds to a particular value of parameter $b^{'}_{IL15}$. To mimic the treatment of employing an anti IL-15 drug, $b^{'}_{IL15}$ is decreased. Also, we assume that the absorption and dynamics of the drug is faster than the dynamics of this subnetwork, and therefore a rapid drop in $b^{'}_{IL15}$ is assumed following administration of anti IL-15 drugs. This drop is modelled by using an inverse rectangular pulse of the following form

**Figure 6:**
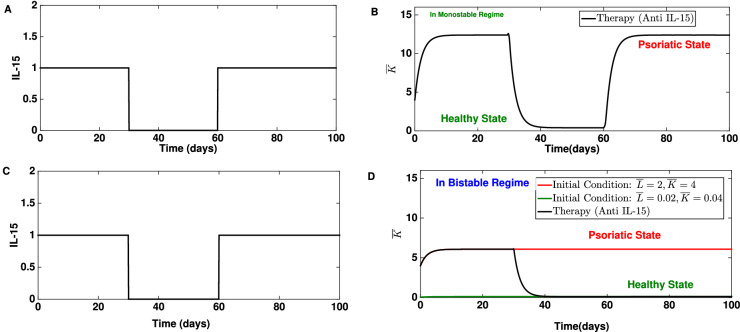
IL-15 signal and treatment of psoriasis using an anti IL-15 drug. (**A**,**C**) An exemplary IL-15 signal before and after using an anti IL-15 drug. The signal has become an inverse rectangular pulse. The signal vanishes completely between the time interval 30–60 days and bounces back to the initial strength immediately after 60 days. (**B**) The dynamics of the keratinocyte population before and after using an anti IL-15 drug. The disease relapses after the effect of the drug vanishe. The model parameters are: $b^{'}_{IL-15}=6, v=0.1,r=0.01, q=0.2, n=3,\mu _L=0.5$ and $\mu _K=0.5$. The default initial condition is: ${\bar{K}}=4$ and ${\bar{L}}=2$. (**D**) Dynamics of the subnetwork before and after applying the anti IL-15 signal shown in (**A**,**C**). Parameters are chosen such that the system could show bistable behavior (point b in two parameter region in Fig. 5D). This demonstrates that after the application of the signal the system would switch from one steady state (psoriatic) to another (healthy state) and remain there. The used model parameter values are: $b^{'}_{IL-15}=3, v=0.1,r=0.01, q=0.05, n=3,\mu _L=0.5$ and $\mu _K=0.5$. The default initial condition is: ${\bar{K}}=4$ and ${\bar{L}}=2$.

2 $$\begin{aligned} S(t) = {\left\{ \begin{array}{ll} 1 &{} \quad t \in [0, t_1] \text { and } t \ge t_2 \\ 0 &{} \quad t \in (t_1, t_2) \\ \end{array}\right. } \end{aligned}$$

where *t* is in days. The time window $(t_1, t_2 )$ corresponds to the duration in which the signal *S* drops completely. The selection of time $t_1$ depends on the dynamics of the subnetwork (and in turn also on the parameter combination), as the signal should fall only after the steady state is reached. The incorporation of an anti IL-15 signal *S* in the system modifies Eq. (14) as follows

3 $$\begin{aligned} \begin{aligned} \frac{{\mathrm {d}}{\bar{K}}}{{\mathrm {d}}t}&= q+b_{IL-15}^{'}S\frac{{\bar{K}}^n }{1+{\bar{K}}^n}-\mu _{K} {\bar{K}} \\ \frac{{\mathrm {d}}{\bar{L}}}{{\mathrm {d}}t}&= r+b_{IL-15}^{'} v \frac{{\bar{K}}^n }{1+{\bar{K}}^n}-\mu _L {\bar{L}} \end{aligned} \end{aligned}$$

This ODE system (Eq. 3) is solved for a parameter combination in which the subnetwork shows bistability (for example the parameter combination in Fig. 5B). The initial condition is chosen such that, eventually, the dynamics of the subnetwork reaches a steady state where ${\bar{K}}$ is high. One such initial condition (${\bar{L}}=2$ and ${\bar{K}}=4$) is shown in Fig. 5E,F. For such an initial condition and parameter combination, the subnetwork would reach the steady state in 20 days (Fig. 5E,F). Therefore, an anti IL-15 signal is used after 20 days, which causes IL-15 to drop fully between 30 and 60 days (Fig. 6A,C). Our results suggest that the keratinocyte population would start decreasing as soon as the signal *S* drops (Fig. 6B,D). A comparison between Figs. 5E and 6D suggests that the keratinocyte population would eventually reach a lower steady state consistent with the initial condition ${\bar{L}}=0.2$ and ${\bar{K}}=0.2$. Interestingly, even if the signal bounces back to the previous level after 60 days, the system would remain in the lower steady state which corresponds to a psoriasis free state. These results demonstrate that anti IL-15 treatment could be a useful treatment for psoriasis. Indeed, IL-15 blockade in a xenograft mouse model has been shown to reduce the severity of psoriasis^38^.

## Discussion

In the present work, the asymptotic behavior of the three subnetworks has been investigated. Based on our numerical analysis, we suggest that increases in the level of cytokines IL-15, IL-17/IL-23 and $\hbox {TNF}_{\alpha }$ would indeed lead to psoriasis but their routes would be different. In the case of a subnetwork involving only $TNF_\alpha$, the hyper-proliferation of keratinocytes would occur monotonically while in subnetworks involving $IL-15$ and IL-23/IL-17 it could occur through a bistable regime. In the case of $\hbox {TNF}_{\alpha }$, psoriasis occurs with gradual increase in keratinocytes, therefore, we believe that an anti-$\hbox {TNF}_{\alpha }$ drug should cure psoriasis gradually. In contrast, in the case of cytokines IL-17/IL-23 and IL-15 the increase in population could happen in an abrupt manner thanks to the bistability. These results are consistent with the observation that IL-17 inhibitors show quicker response and better efficacy compared to anti-$\hbox {TNF}_\alpha$ biologics^21,22^. However, based on this study we cannot attribute these observations to bistable behaviour although this warrants further investigation. In addition, here it is demonstrated that the bistable behavior could be an advantage for developing new treatments for psoriasis. The suggested treatment could be a strategy through which first the severity of the psoriasis could be lessened by appropriately perturbing the system even if the clearance of psoriasis is not achievable. Once the severity is reduced, medicines can be prescribed which would reduce or at least maintain the achieved low severity. Our results also suggests that targeting two or more cytokines at a time could be an effective strategy, which parallels movements to advocate bispecific agents^39^. Interestingly, dual targets have previously been trialled for psoriasis (IL-17 with $\hbox {TNF}_{\alpha }$)^40^. Based, on our results we argue that targeting IL-15 and IL-17/IL-23 would be more effective than targeting $\hbox {TNF}_{\alpha }$.

One of the limitations of this work is that only three subnetworks of the full network described in Ref.^18^ are studied. Another limitation is, here, results are shown only for a small region of multidimensional parameter space. Further, while investigating the treatment of psoriasis via ‘anti-IL-15’ treatment only one type of signal was assumed to have perturbed the system. To overcome these limitations the model should be expanded upon in a number of ways in the future. Firstly, other important components for psoriasis pathogenesis should be incorporated. These would include key immune cells such as macrophages, neutrophils and natural killer cells. Additionally, future models should investigate other cytokines that are implicated in psoriasis, such as IL-22. In our model, for simplicity, IL-17 represents all members of its family. Future work should address the role of IL-17 subtypes. Model results should also be compared with the available experimental data. In future, by exploring patient-specific models and predicting their response to treatments, one could aim for personalized medicine.

## Methods

In^18^, we synthesised existing knowledge into a schematic of the cytokine-mediated feedback network between keratinocytes, T-cells and dendritic cells. This schematic is reproduced in Fig. 1A. In order to mediate a tractable analysis of the dynamics of this network, in the context of treatment for psoriasis, we highlight three sub-networks of interest, mediated by one or two cytokines (IL-23/IL-17, $\hbox {TNF}_\alpha$ and IL-15) in Fig. 1B–D. The dynamics of these sub-networks is the focus of the current study.

### The mathematical modelling framework

The population dynamics of the considered cell types is modelled using ordinary differential equations (ODEs) as proposed in our previous general model for psoriasis pathogenesis^18^.

4 $$\begin{aligned} \frac{dx}{dt} = a_x + b_{s}(x)-\mu _x x \end{aligned}$$

where $a_x$ represents a basal rate of increase in the cell population *x* due to migration or differentiation factors, which is assumed to be independent of the current population *x*. The third term ($\mu _x$) represents the decay in cell population levels (for example apoptosis independent of modelled cytokines). The second term represents a net birth rate which encompasses the cytokine-mediated effects of (1) differentiation/maturation, (2) proliferation and (3) apoptosis. We assume a saturation in the birth rate, dependent upon the cell population (*x*) and therefore use either of following two forms, to account for enhancing or diminishing effects of cytokines:

(i) if $b_s$ increases with an increase in the cell population *x*
  $$\begin{aligned} b_s=b_{x}\frac{x^n}{k_x^n+x^n} \end{aligned}$$
(ii) if $b_s$ decreases with an increase in the cell population
  $$\begin{aligned} b_s=b_{x}\frac{k_x^n}{k_x^n+x^n}. \end{aligned}$$
  Here, $b_x$ represents the saturated rate of change due to change in the population *x*. The parameter $k_x$ can be interpreted as the fraction of the population *x* required to achieve half the maximal increase/decrease in *x* due to the population *x*. The parameter *n* is the Hill coefficient, which determines the slope of the function $b_s$.

In the following sections we introduce specific forms of Eq. (4) for the three subnetworks of interest.

#### $\text {TNF}_{\alpha }$ sub-network model

Figure 1B details the interactions between cells driven by $\text {TNF}_{\alpha }$. Using the general system (Eq. 4), these interactions are modelled using the following set of ODEs:

5 $$\begin{aligned} \begin{aligned} \frac{{\mathrm {d}}L}{{\mathrm {d}}t}&= a_L-\mu _{L} L \\ \frac{{\mathrm {d}}D}{{\mathrm {d}}t}&= a_{D}+b_{TNF_{\alpha }} \left( \frac{D^n }{k_D^n+D^n}\frac{L^n }{k_L^n+L^n}\frac{K^n}{k_K^n+K^n} \right) -\mu _{D} D\\ \frac{{\mathrm {d}}K}{{\mathrm {d}}t}&= a_{K}+b_{TNF_{\alpha }}\left( \frac{k_K^n }{k_K^n+K^n}\frac{k_D^n}{k_D^n+D^n}\frac{k_L^n}{k_L^n+L^n}\right) -\mu _{K}K \end{aligned} \end{aligned}$$

where *L*, *D* and *K* denote the current population of T cells, matured dendritic cells and keratinocytes, respectively. The parameter $b_{TNF _{\alpha }}$ represents the maximum rate of increase in *D* and *K* due to the effect of the cytokine $\hbox {TNF}_{\alpha }$ which is secreted from all three types of cells. The parameter $k_D$ denotes the population of dendritic cells required for half of the maximum effect *D* can achieve on *K* or *D*. Similarly, $k_K$ (or $k_L$) represents the population of keratinocytes (or T-cells) required for half of the maximum effect *K* (or *L*) can have on *D* or *K*.

From Eq. (5), it is obvious that the dynamics of the T-cells is independent of the dynamics of the dendritic cells and keratinocytes. Therefore, a quasi-steady state scenario for T-cells can be assumed and the ODE system, Eq. (5), will be predominantly governed by the following two dimensional system

6 $$\begin{aligned} \begin{aligned} \frac{{\mathrm {d}}D}{{\mathrm {d}}t}&= a_{D}+ b_{TNF_{\alpha }} \left( \frac{{L^*}^n }{k_L^n+{L^*}^n}\frac{D^n }{k_D^n+D^n} \frac{K^n}{k_K^n+K^n} \right) -\mu _{D} D\\ \frac{{\mathrm {d}}K}{{\mathrm {d}}t}&= a_{K}+ b_{TNF_{\alpha }} \left( \frac{k_K^n }{k_K^n+K^n}\frac{k_D^n}{k_D^n+D^n}\frac{k_L^n}{k_L^n+{L^*}^n}\right) -\mu _{K}K \end{aligned} \end{aligned}$$

where $L^*=\frac{a_L}{\mu _L}$ represents the steady state population of T-cells.

In order to further reduce the complexity of this and subsequent systems, we non-dimensionalise these equations. Assuming that ${\bar{K}}=\frac{K}{k_k}$, ${\bar{L}}=\frac{L^*}{k_L}$ and ${\bar{D}}=\frac{D}{k_D}$, the Eq. (6) can be represented by the dimensionless system

7 $$\begin{aligned} \frac{{{\text{d}}\bar{D}}}{{{\text{d}}t}} = & p + b^{\prime}_{{TNF}} \left( {\frac{{\bar{L}^{n} }}{{1 + \bar{L}^{n} }}\frac{{\bar{D}^{n} }}{{1 + \bar{D}^{n} }}\frac{{\bar{K}^{n} }}{{1 + \bar{K}^{n} }}} \right) - \mu _{D} \bar{D} \\ \frac{{{\text{d}}\bar{K}}}{{{\text{d}}t}} = & q + b^{\prime}_{{TNF}} u\left( {\frac{1}{{1 + \bar{K}^{n} }}\frac{1}{{1 + \bar{D}^{n} }}\frac{1}{{1 + \bar{L}^{n} }}} \right) - \mu _{K} \bar{K} \\ \end{aligned}$$

where $$p=\frac{a_D}{k_D}, b_{TNF}^{'}=\frac{b_{TNF_{\alpha }}}{k_D}, q=\frac{a_{K}}{k_K}$$, and $$u=\frac{k_D}{k_K}$$

#### IL-23/IL-17 sub-network model

The cytokines IL-23 and IL-17 are currently used as targets for the treatment of psoriasis^20–22^, and are therefore crucial components of our study. Figure 1A,C demonstrate that these cytokines form a feedback loop between dendritic cells and T-cells, but that there is no direct link to keratinocytes. Rather, the effect on keratinocytes is mediated by a network of connections involving other cytokines. In order to simplify this scenario, we postulate a net effect of the IL-23/IL-17 axis on the keratinocyte populations. We consider that this net effect can either be activating or inhibiting, which gives rise to the following two cases:

Case (1): The net effect of *D* and *L* on *K* is activating

8 $$\begin{aligned} \begin{aligned} \frac{\mathrm {d}L}{\mathrm {d}t}&= a_L+b_{IL23}\left( \frac{K^n }{k_K^n+K^n}\frac{D^n}{k_D^n+D^n}\right) -\mu _{L} L \\ \frac{\mathrm {d}D}{\mathrm {d}t}&= a_{D}+b_{IL17}\frac{L^n }{k_L^n+L^n} -\mu _{D} D\\ \frac{\mathrm {d}K}{\mathrm {d}t}&= a_{K}+ b_{net}\frac{A^n }{k_A^n+A^n}-\mu _{K} K \end{aligned} \end{aligned}$$

where $A=D+L$. *L*, *D* and *K* represent the population of modelled cells as for the previous subnetworks. Also, parameters $b_{IL23}$, $b_{IL17}$ and $b_{net}$ can be defined as for the corresponding terms in the previous subnetworks. The dimensionless form of Eq. (8) becomes

9 $$\begin{aligned} \frac{\text{d}{\bar{L}}}{\mathrm {d}t}&=r+ b_{IL23}^{'} \left( \frac{{\bar{K}}^n }{1+{\bar{K}}^n}\frac{{\bar{D}}^n}{1+{\bar{D}}^n}\right) -\mu _{L} {\bar{L}} \\ \frac{\text{d}{\bar{D}}}{\mathrm {d}t}&= p+b_{IL17}^{'} \frac{{\bar{L}}^n }{1+{\bar{L}}^n} -\mu _{D} {\bar{D}}\\ \frac{\text{d}{\bar{K}}}{\mathrm {d}t}&=q+b_{net}^{'}\frac{{\bar{A}}^n }{k_A^n+{\bar{A}}^n}-\mu _{K} {\bar{K}} \end{aligned}$$

where ${\bar{L}}=\frac{L}{k_L}$, ${\bar{D}}=\frac{D}{k_D}$, ${\bar{K}}=\frac{K}{k_K}$, ${\bar{A}}={\bar{D}}+{\bar{L}}$, $r= \frac{a_L}{k_L}, b_{IL23}^{'}=\frac{b_{IL23}}{k_L}, b_{IL17}^{'}=\frac{b_{IL17}}{k_D}$ and $b_{net}^{'}= \frac{b_{net}}{k_K}$. Parameters p and q, are defined same as in the previous subnetwork.

Case (2): The net effect of *D* and *L* on *K* is inhibitory. The population dynamics of the subnetwork is

10 $$\begin{aligned} \frac{\mathrm {d}L}{\mathrm {d}t}&= a_L+b_{IL23}\left( \frac{K^n }{k_K^n+K^n}\frac{D^n}{k_D^n+D^n}\right) -\mu _{L} L \\ \frac{\mathrm {d}D}{\mathrm {d}t}&= a_{D}+b_{IL17}\frac{L^n }{k_L^n+L^n} -\mu _{D} D\\ \frac{\mathrm {d}K}{\mathrm {d}t}&= a_{K}+ b_{net}\frac{k_A^n }{k_A^n+A^n}-\mu _{K} K \end{aligned}$$

where $A=D+L$. Other terms have the same meaning as in case (1). The dimensionless representation is

11 $$\begin{aligned} \frac{\text{d}{\bar{L}}}{\mathrm {d}t}&= r+b_{IL23}^{'} \left( \frac{{\bar{K}}^n }{1+{\bar{K}}^n}\frac{{\bar{D}}^n}{1+{\bar{D}}^n}\right) -\mu _{L} {\bar{L}} \\ \frac{\text{d}{\bar{D}}}{\mathrm {d}t}&= p+b_{IL17}^{'} \frac{{\bar{L}}^n }{1+{\bar{L}}^n} -\mu _{D} {\bar{D}}\\ \frac{\text{d}{\bar{K}}}{\mathrm {d}t}&= q+ b_{net}^{'}\frac{k_A^n }{k_A^n+{\bar{A}}^n}-\mu _{K} {\bar{K}}\end{aligned}$$

where ${\bar{L}}=\frac{L}{k_L}$, ${\bar{D}}=\frac{D}{k_D}$, ${\bar{K}}=\frac{K}{k_K}$ and ${\bar{A}}={\bar{D}}+{\bar{L}}$. *p*, *q*, *r*, $b_{IL23}^{'}$, $b_{IL17}^{'}$, and $_{net}^{'}$ are defined same as in Case (1).

#### IL-15 sub-network model

IL-15 arises as an interesting cytokine as it mediates self-activation of keratinocytes (Fig. 1A). In addition, it promotes the production of cytokines such as IFN-$\gamma$, $TNF_{\alpha }$ and IL-17 and therefore a potentially important mediator of the psoriasis phenotype^40^ . The IL-15 sub-network (Fig.  1D) is modelled as follows:

12 $$\begin{aligned} \frac{\mathrm {d}L}{\mathrm {d}t}&= a_L+b_{IL-15}\left( \frac{K^n }{k_K^n+K^n}\frac{D^n}{k_D^n+D^n}\right) -\mu _{L} L \\ \frac{\mathrm {d}D}{\mathrm {d}t}&= a_{D} -\mu _{D} D\\ \frac{\mathrm {d}K}{\mathrm {d}t}&= a_{K}+b_{IL-15}\left( \frac{K^n }{k_K^n+K^n}\frac{D^n}{k_D^n+D^n}\right) -\mu _{K} K \end{aligned}$$

where *L*, *D* and *K* have the same meaning as in the previous subnetworks. The parameter $b_{IL-15}$ represents the maximum rate of increase in T-cells and keratinocyte population due to changes in the modelled cell types; it reflects the effect of changing the level of IL-15. Note that changes in *D* and *K* will lead to changes in the level of IL-15 as it is secreted from *D* and *K*. The parameter $k_K$ can be described as the population of keratinocytes required for half of the maximum effect *K* can achieve on *K* or *L*. Similarly, $k_D$ can be defined as the population of dendritic cells required for half of the maximum effect *D* can have on *L* or *K*.

In Eq. (12), the dynamics of the dendritic cells is independent of the dynamics of the T-cells and keratinocytes. Therefore, a quasi-steady state scenario is assumed for dendritic cells and the ODE system (Eq. 12) will be largely governed by the following two dimensional system

13 $$\begin{aligned} \frac{\mathrm {d}L}{\mathrm {d}t}&= a_L+b_{IL-15}D^{*} \frac{K^n }{k_K^n+K^n}-\mu _{L} L\\ \frac{\mathrm {d}K}{\mathrm {d}t}&= a_{K}+b_{IL-15}D^{*}\frac{K^n }{k_K^n+K^n}-\mu _{K} K \end{aligned}$$

with $D^{*}= \frac{\left( \frac{a_{D}}{\mu _{D}}\right) ^n}{k_D^n +\left( \frac{a_{D}}{\mu _{D}}\right) ^n}$. The corresponding dimensionless system is given by

14 $$\begin{aligned} \frac{\text{d}{\bar{K}}}{\mathrm {d}t}&= q+b_{IL-15}^{'} \frac{{\bar{K}}^n }{1+{\bar{K}}^n}-\mu _{K} {\bar{K}} \\ \frac{\text{d}{\bar{L}}}{{\mathrm {d}}t}&= r+b_{IL-15}^{'} v \frac{{\bar{K}}^n }{1+{\bar{K}}^n}-\mu _L {\bar{L}} \end{aligned}$$

where ${\bar{K}}=\frac{K}{k_K}$ and ${\bar{L}}=\frac{L}{k_L}$ are dimensionless quantities and denote the relative population of keratinocytes and T-cells, respectively (relative to $k_K$). The $b_{IL-15}^{'}=\frac{b_{IL-15}D^{*}}{k_K}, v=\frac{k_K}{k_L}$. Parameters *q* and *r* are defined same as in case of subnetwork involving $TNF_{\alpha }$.

### Model parameters

The parameters of the system, and their default values are given in Table 1. The rate of apoptosis of T-cells ($\mu _L$) has been estimated as 0.5 day$^{-1}$ during their response to Lymphocytic Choriomeningitis Virus^41^. The rate of apoptosis of cutaneous dendritic cells ($\mu _D$) has been observed to be approximately 0.2 day$^{-1}$^42^. The rate of apoptosis of keratinocytes ($\mu _K$ ) is unknown therefore given these two estimates, we choose a similar value, 0.5 day$^{-1}$, for that of keratinocytes.

In the subsequent section, we use the dynamics of the $\hbox {TNF}_\alpha$ subnetwork to determine further parameters, based on the existence of high and low keratinocyte states.

**Table 1:** Parameters and their default values.

Parameter	Default value
*p*	0.01 day$^{-1}$
*q*	0.1 day$^{-1}$
*r*	0.01 day$^{-1}$
*u*	10
*v*	0.1
$\mu _K$	0.5 day$^{-1}$
$\mu _L$	0.5 day$^{-1}$
$\mu _D$	0.2 day$^{-1}$
$b_{TNF}^{'}$	1 day$^{-1}$
$b_{IL23}^{'}$	10 day$^{-1}$
$b_{IL17}^{'}$	10 day$^{-1}$
$b_{IL15}^{'}$	4 day$^{-1}$
$b_{net}^{'}$	5 day$^{-1}$
$L^*$	0.1

## Supplementary information

Supplementary material 1

## Data Availability

The datasets generated for this study can be found in the manuscript and supplementary Data.
