# Supplementary material for: Role of subnetworks mediated by TNFα, IL-23/IL-17 and IL-15 in a network involved in the pathogenesis of psoriasis
Source: Sci Rep. 2021 Jan 26;11:2204. doi: 10.1038/s41598-020-80507-7 (PMC7838322; doi:10.1038/s41598-020-80507-7)
Supplement: Supplementary file 1 — Supplementary material 1 [file 41598_2020_80507_MOESM1_ESM.pdf]

Role of subnetworks mediated by TNF$_\alpha$, IL-23/IL-17 and IL-15 in a network involved in the pathogenesis of psoriasis

Rakesh Pandey[1,+*], Yusur Al-Nuaimi[2], Rajiv Kumar Mishra[3,++], Sarah K. Spurgeon[4], and Marc Goodfellow[1,5,6,7*]

[1]College of Engineering, Mathematics and Physical Sciences, University of Exeter, Exeter, UK
[2]Department of Dermatology, Royal Devon and Exeter Hospital, Exeter, UK
[3]Institute of Science and Technology Austria, Am Campus 1, Klosterneuburg, Austria
[4]Department of Electronic and Electrical Engineering, University College London, London, UK
[5]Living Systems Institute, University of Exeter, Exeter, UK
[6]Wellcome Trust Centre for Biomedical Modelling and Analysis, University of Exeter, Exeter, UK
[7]EPSRC Centre for Predictive Modelling in Healthcare, University of Exeter, Exeter, UK
[*]rakeshpandey@bhu.ac.in and M.Goodfellow@exeter.ac.uk
[+]Present address: Bioinformatics Section, Mahila Mahavidyalay, Banaras Hindu University, Varanasi, India
[++]Present address: Premas Biotech Private Ltd. Manesar, Gurugram, India

ABSTRACT

1 Figure S1

2 Figure S2

3 Figure S3

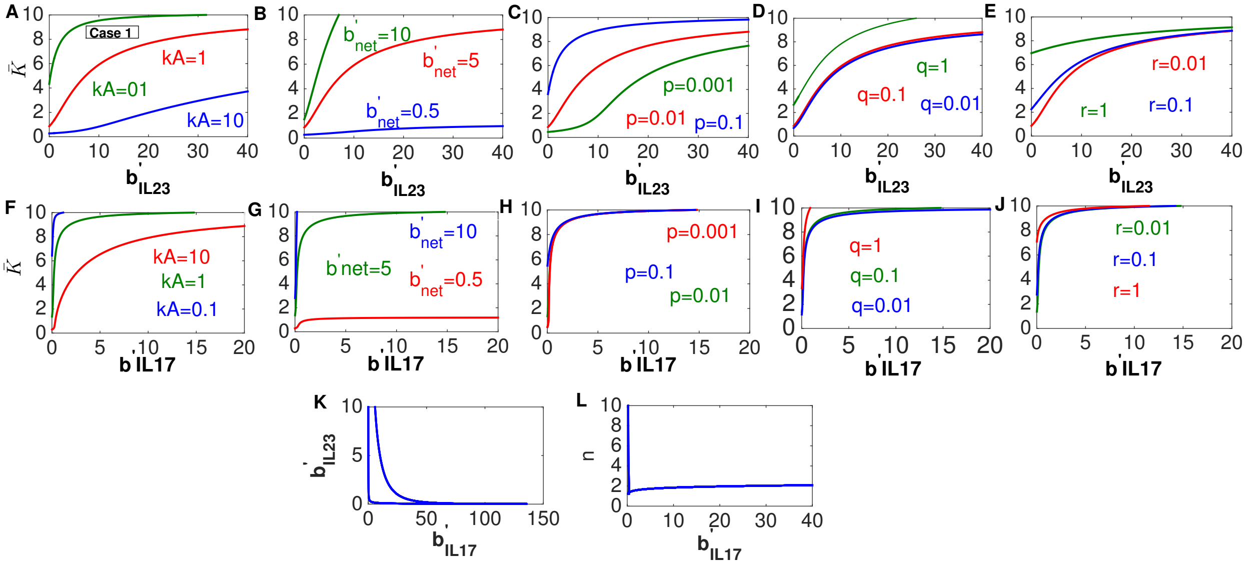

Figure S1. Signal response curves for the subnetwork formed by IL-23 and IL-17 for Case 1. Effects of changes in model parameters on the population of keratinocytes are displayed. Here, the combined level of IL-23 and IL-17 is represented by b_{net}. The default parameters are: $b'_{IL23} = 1$, $b'_{IL17} = 0.01$, $b_{net} = 5$, $p = 0.01\ day^{-1}$, $q = 0.1\ day^{-1}$, r=0.01, n=1, $\mu_L = 0.5\ day^{-1}$, $\mu_K = 0.5\ day^{-1}$, $\mu_D = 0.2\ day^{-1}$, and $k_A = 1$. The initial condition is $\bar{L} = \bar{D} = 2$ and $\bar{K} = 4$

.

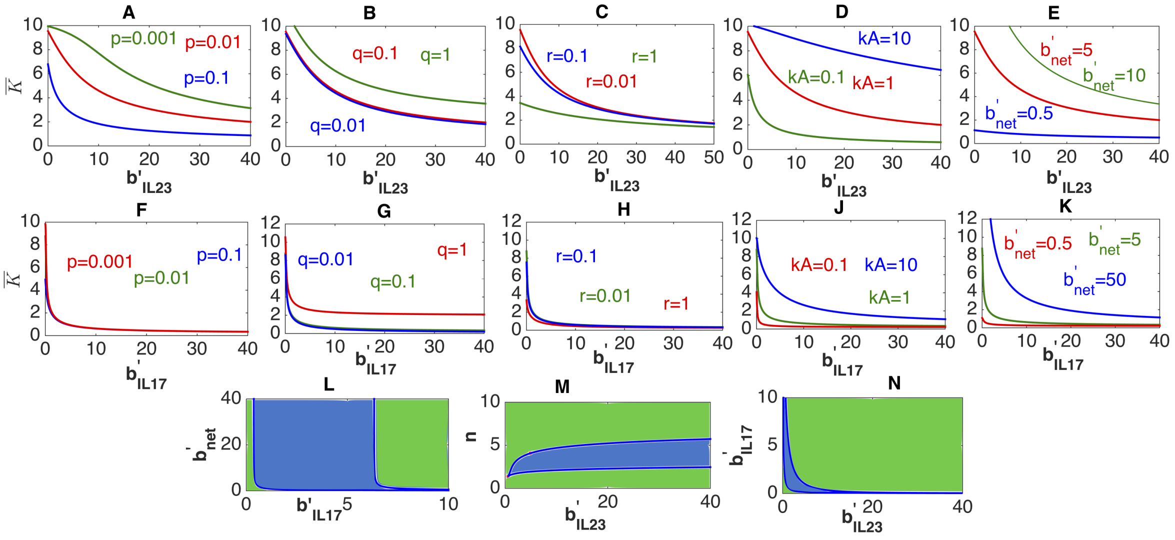

Figure S2. Signal response curves for the subnetwork formed by IL-23 and IL-17 for Case 2. Parameters are same as in Figure S1.

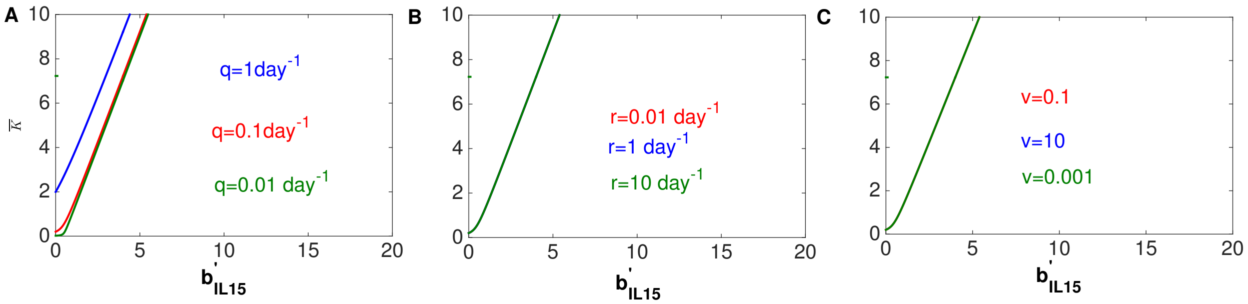

Figure S3. Signal response curves for the subnetwork formed by IL-15. The model parameters used here are same as in Figure 5.
